# Cascading effects of temperature alterations on trophic ecology of European grayling (*Thymallus thymallus*)

**DOI:** 10.1038/s41598-019-55000-5

**Published:** 2019-12-04

**Authors:** Szymon Smoliński, Adam Glazaczow

**Affiliations:** 1 Department of Fisheries Resources, National Marine Fisheries Research Institute, Kołłątaja 1, 81-332 Gdynia, Poland; 2 Demersal Fish Research Group, Institute of Marine Research, P.O. Box 1870 Nordnes, 5817 Bergen, Norway; 3 Department of Systematic Zoology, Adam Mickiewicz University, Uniwersytetu Poznańskiego 6, 61-614 Poznan, Poland

**Keywords:** Phenology, Freshwater ecology, Phenology, Freshwater ecology

## Abstract

The aims of this project were to study: diet composition, food selectivity and the phenology of different prey items in grayling’s (*Thymallus thymallus*) diet. It was hypothesized, that alterations in mayfly emergence, caused by reservoir-induced thermal changes, have consequences for trophic ecology of drift-feeding fish. Sampling of fish and macroinvertebrates were conducted in two closely located rivers, one human-modified and the other an undisturbed river. Grayling preyed mainly on aquatic insects, but only mayflies were preferred. Seasonal changes of the fish diet were observed, and air temperature is considered a predictor of prey occurrence with different time lags, depending on the biology of the organisms. Significant differences in the abundances and probability of mayfly occurrence between two studied rivers were shown. The observed phenological shift suggests that distorted environmental cues were experienced by the Ephemeroptera in the modified river. The “lost generation” of insects which failed to complete development became a new food for fish. The results presented indicate that reservoir-induced thermal alterations in the rivers, similarly to climate change, can lead to a chain of consequences in the ecosystems. Taking into consideration the projected climate scenarios, further monitoring and forecasting of these effects are considered an important step for future mitigating actions and adaptive management of water resources.

## Introduction

River temperature is an important physical characteristic of water quality and it affects many aspects of freshwater ecology^1^. Predicted increases in mean global air temperatures^2^ will translate directly into changes in the temperature of running waters^3^. A primary concern for wild species is the rapid rate of these changes, weakening the evolutionary response of organisms^4^. Similar abrupt alterations of thermal regimes in freshwater ecosystems can be observed in watercourses, which have been regulated in the recent past by artificial reservoirs^5^. The shallow basins with large surface areas raise their temperature during summer^6^. The increase of temperature, caused by the presence of reservoirs or by global warming may have a similar marked impact on the ecosystem, both by direct and indirect effects on different ecosystem components, e.g. macroinvertebrate and fish communities.

In the natural river systems, water temperature serves as an environmental cue used by insects to predict future environmental conditions and synchronize different life phases with favourable periods^7^. However, the abruptly changing temperature of the water below the dam may distort the quality of information given by this environmental cue that insects use to make developmental decisions and to tune their phenology^8,9^. If this rapid environmental alteration triggers organisms to pursue the maladaptive life-cycle decision, this can lead to the developmental trap, a special case of evolutionary trap^9–12^, e.g. prolonged summer diapause resulting in too late onset of the last generation in autumn, and reduced success of emergence due to the suboptimal water and air temperature^8^.

Responses by individual species to climate change are connected through interactions with others at the same or adjacent trophic levels^13^, therefore reduced success of the emergence of insects and their higher mortality may have a further impact on fish species. By increased availability, they may become a new source of food for these predators, which change their foraging behaviour. Such an effect of altered river conditions propagating through tail-water food webs is still relatively rarely reported and the consequences of altering macroinvertebrate drift concentration and composition for drift-feeding fishes remain largely unknown^14,15^.

One of the drift-feeding fish is the European grayling (*Thymallus thymallus*), which occurs in the central, northern and north-eastern Europe^16^. Grayling use a sit-and-wait foraging tactic in the rivers and their capture rate is positively correlated with prey density^17^. Drift feeding is considered to be a strategy in energy optimization, whereby fish select locations that provide high energy intake whilst minimizing their energy expenditure^18^. The main food of grayling is aquatic invertebrates, complemented by terrestrial invertebrates and occasionally small fish^19^. The species feeds more often on benthos such as Trichoptera larvae, molluscs and crustaceans, than on surface insects^20^. Mayflies were in the diet of the most fish, although in some cases they were only a fraction of a percent and where they were most numerous (e.g. about 40% of all diet items in the river Shipot, Western Ukraine), nymphs were mostly eaten^21^.

**Figure 1:**
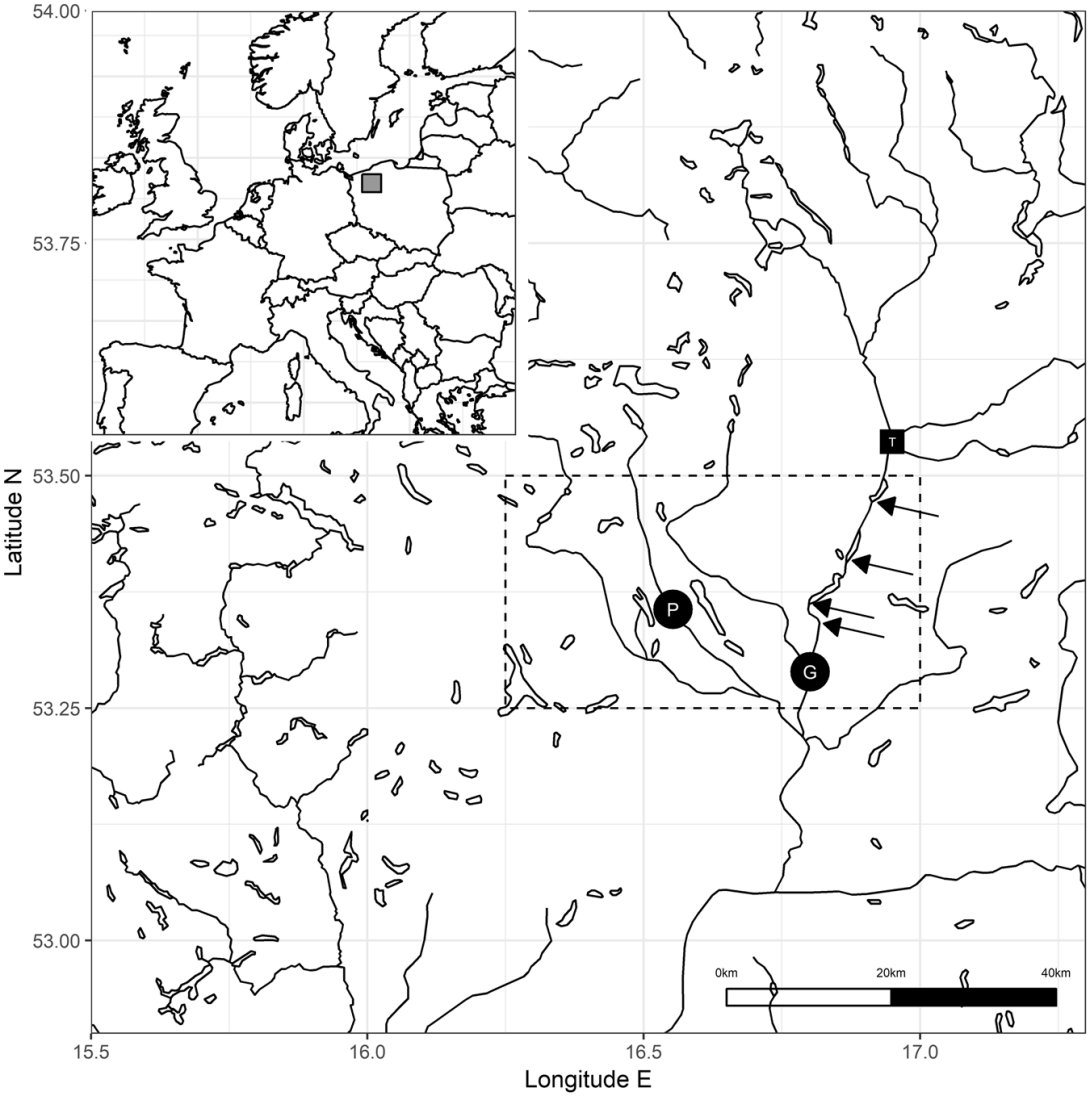
Fish sampling area (dark points: G-Gwda; P-Pilawa) with indicated area over which gridded climatic data were aggregated (dashed line). Location of four major artificial dams are marked with arrows. Location above dams in which additional temperature data were recorded is marked with dark square (T).

A previous study conducted on the river Gwda (Fig. 1) showed that dams and reservoirs built along the river significantly altered water temperature^8^. Moreover, the study has shown, that increased temperature in summer delayed the emergence of the last generation of mayfly for colder months (October and November) and because the success of the insect emergence is positively related to the water temperature, it is extremely low in that period. Thus, the altered insect phenology on the Gwda resulted in a largely lost generation of the mayflies unable to take-off for moulting and mating^8^. Current observations showed, that these mayfly subimagines (winged stage) were numerously eaten by grayling in the river Gwda, suggesting cascading effects of water temperature disruptions on the development of macroinvertebrates, which in turn has an impact on predator-prey relations in the environment and drift-feeding fish.

Given the complexity of many ecosystems, we often lack profound knowledge of the potential climatic effects on feeding ecology of freshwater fish^22^. Improved understanding, monitoring, and forecasting of these effects are thus crucial for researchers, policymakers and biodiversity managers^23^. The disturbances in temperature regimes observed in the human-modified watercourses form an opportunity to learn about potential mechanisms of these changes on aquatic ecosystems, which according to the projected climate scenarios^2,24^, may occur in the future also in natural rivers and hence affect predator-prey dynamics^17^.

For these reasons, we conducted a study on European grayling feeding ecology in one natural and one human-modified river located in central Europe. We hypothesized that modified environmental conditions can cause cascading effects on river ecosystems, through the reduced success of mayfly emergence, which in consequence can increase the availability of these prey items and affect the trophic ecology of grayling. We expected that reduced success of mayflies’ emergence in the thermally disturbed river will contribute to their higher abundance, contribution, and frequency of occurrence in the composition of the grayling diet and that this taxon constitutes food preferred by grayling. Additionally, we wanted to check the predictability of the occurrence of different groups of diet items in the fish stomachs with air temperature as a predictor. We hypothesized, that because of altered development phenology and reduced success of emergence, mayflies (unlike other groups) will appear with higher probability in the diet of fish in the modified river (Gwda) under the same ambient air temperature conditions than in the closely located natural watercourse (Pilawa). Thus, the aims of this paper were to investigate: i) diet composition and ii) food selectivity of grayling, and iii) the phenology of the emergence of individual prey groups in the diet of fish, using data collected in the years 2005–2016.

## Results

### Water temperature

Conducted GLM tests showed significant (p < 0.05) differences in the water temperature above and below the artificial dams located on the river Gwda (Table S1, Fig. S1). On average, water below the dams was warmer than above. The increased temperature was observed especially in the summer months (water temperature was higher below dams by 1.92 °C in August and by 0.78 °C in September). Moreover, accumulated degree days in the period 1^st^ of August to 31^st^ of November were higher below dams by 90 °C·days (Fig. S2).

### Diet composition and food selectivity of grayling

**Figure 2:**
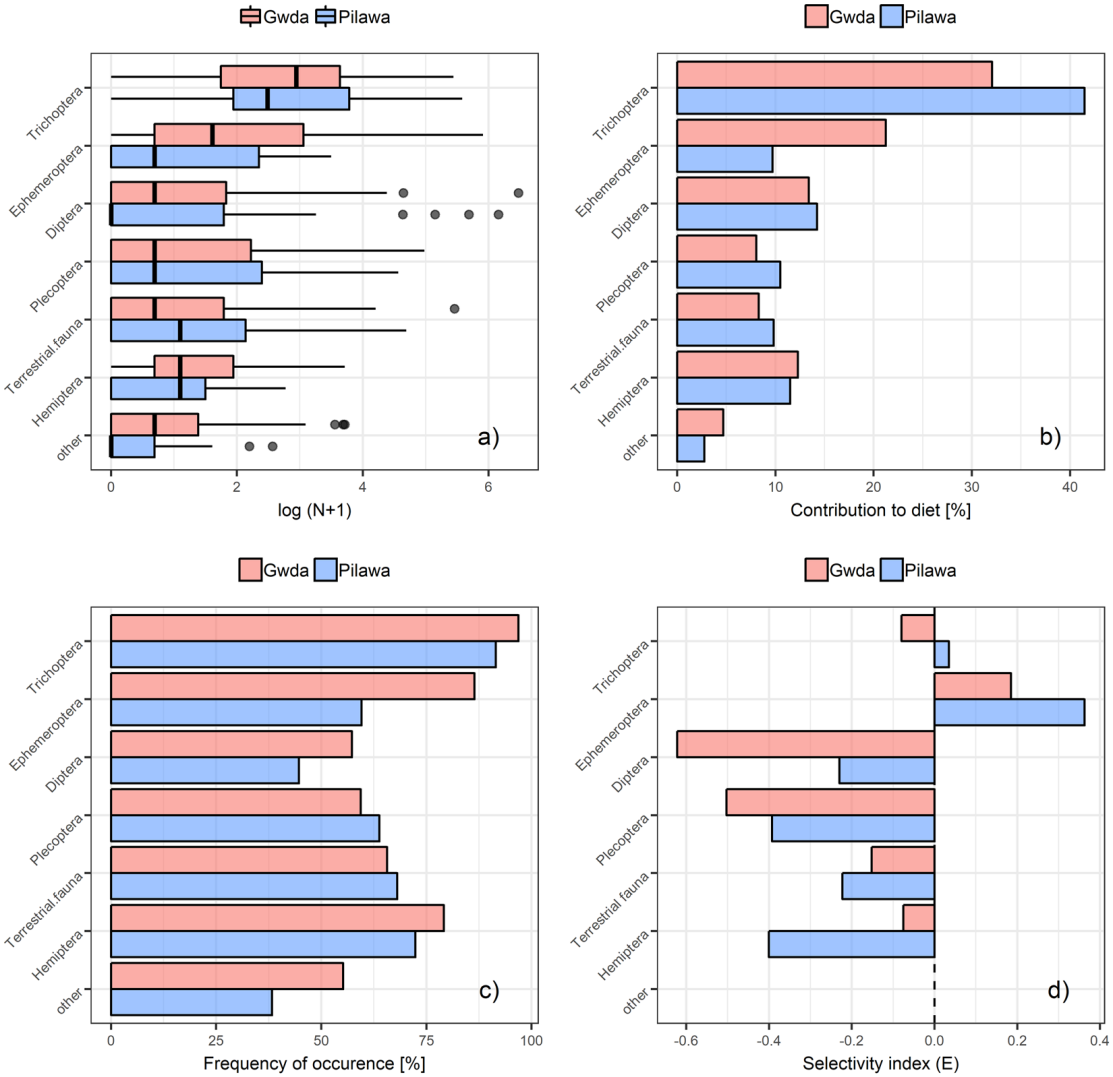
Abundance of particular prey group in the diet of European grayling *Thymallus thymallus* (**a**), mean contribution to all diet items (**b**), frequency of occurrence in all fish stomachs (**c**) and mean Ivlev’s selectivity index E (**d**). No selectivity index was calculated for the group ‘other’.

The grayling diet consisted of about a dozen different taxa, both water and terrestrial animals. Only 2–3 or exceptionally 4 taxons were eaten to any large extent by a single fish. In the stomachs were predominantly: *Brachycentrus subnubilus* (Trichoptera), Simulium (Diptera), *Baetis liebenauae* (Ephemeroptera), *Leuctra fusca*, *Taeniopteryx nebulosa* (Plecoptera), and *Aphelocheirus aestivalis* (Hemiptera). Trichoptera and Ephemeroptera were the most abundant groups of prey eaten by grayling (Fig. 2a) with a contribution of 35.2% and 17.5%, respectively (Fig. 2b). Trichoptera were found in the fish stomachs with the highest frequency (95.1%), followed by Ephemeroptera (77.6%) and Hemiptera (76.9%) (Fig. 2c). Terrestrial fauna in the fish stomachs was mainly represented by Araneidae and univoltine insects, like Lepidoptera and Coleoptera. Among the others, less abundant items (contribution to diet <5%), we observed different preys like Oligochaeta, Hirudinea, Odonata, Coleoptera, crustaceans (*Gammarus* and *Asellus*), which were the most abundant between them, and molluscs or even fish roe. Comparison of the composition of diet content and macroinvertebrate drift in 2008–2010 showed clearly, that among the main groups of prey eaten by grayling only Ephemeroptera were chosen with high preference. The mean values of Ivlev’s selectivity index calculated for Ephemeroptera were 0.18 and 0.36 for Gwda and Pilawa, respectively (Fig. 2d). For Trichoptera calculated selectivity values were close to zero, while other groups of organisms were avoided by fish to some extent.

### Abundance of the main groups of prey in the diet

**Figure 3:**
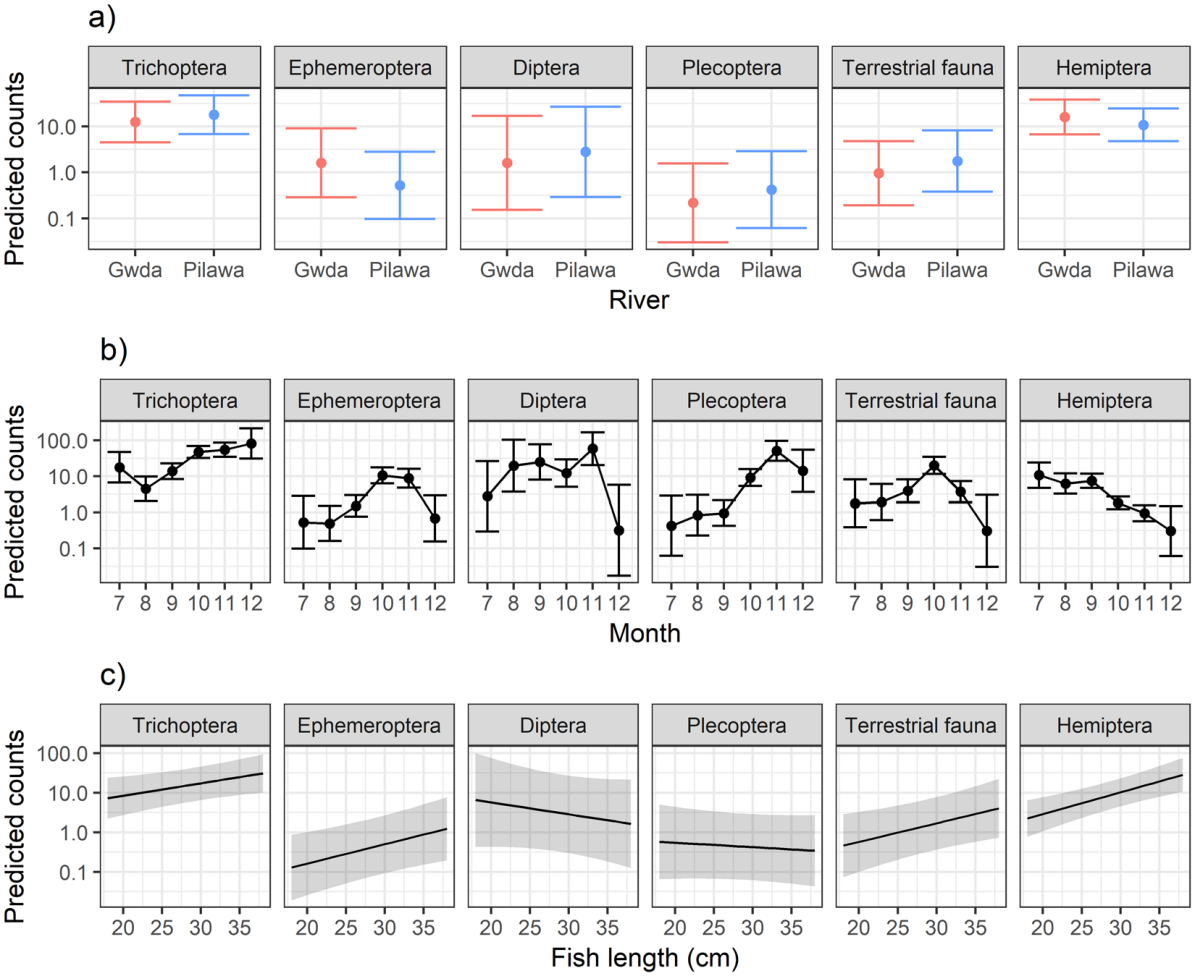
Effects of river (**a**), month (**b**) and fish length (**c**) on counts of main diet groups of grayling predicted by GLM models with negative binomial distribution. Dots and lines show predicted values, while error bars and shaded areas present confidence intervals. Y-axis is presented in logarithmic scale.

Conducted GLM tests showed significant (p < 0.05) differences in the abundances of Ephemeroptera and Plecoptera in the diet of fish from the two studied rivers (Fig. 3a, Table 1). Abundance of Ephemeroptera was higher and abundance of Plecoptera was lower in the fish stomachs from the Gwda than from the Pilawa. There were no differences in abundances between these two rivers for other groups of prey. A strong seasonal pattern was visible in the grayling diet (Fig. 3b) and the effect of the month was statistically significant for all the groups of diet (Table 1). The fish length was a significant predictor for the abundance of Trichoptera, Ephemeroptera, terrestrial fauna and Hemiptera (Table 1). For all these groups, relationships between fish length and the number of counts in the stomach content were positive (Fig. 3c).

**Table 1:** Summary of GLM negative binomial models fitted to the abundance data of main groups of grayling diet.

Group	Term	LR Chisq	Df	P
Trichoptera	river	2.98	1	0.084
Trichoptera	month	49.31	5	<0.001
Trichoptera	length	3.94	1	0.047
Ephemeroptera	river	13.96	1	<0.001
Ephemeroptera	month	55.59	5	<0.001
Ephemeroptera	length	9.71	1	0.002
Diptera	river	1.16	1	0.280
Diptera	month	16.04	5	0.007
Diptera	length	0.32	1	0.569
Plecoptera	river	4.36	1	0.037
Plecoptera	month	100.13	5	<0.001
Plecoptera	length	0.28	1	0.599
Terrestrial fauna	river	3.48	1	0.062
Terrestrial fauna	month	48.34	5	<0.001
Terrestrial fauna	length	4.39	1	0.036
Hemiptera	river	3.61	1	0.058
Hemiptera	month	96.69	5	<0.001
Hemiptera	length	16.36	1	<0.001

LR Chisq - likelihood-ratio chi square, Df - degrees of freedom, P – significance level.

**Figure 4:**
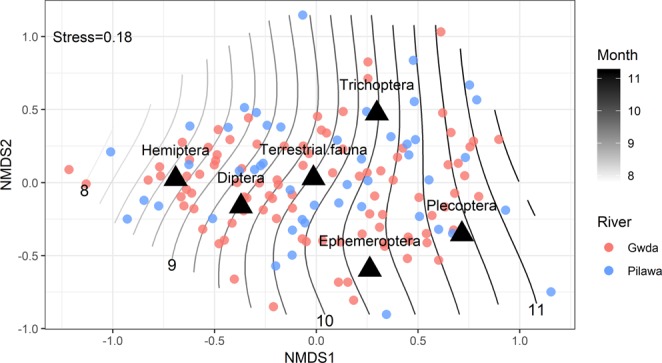
Non-metric multidimensional scaling (NMDS) ordination of grayling diet composition based on Bray-Curtis dissimilarity matrix (dots) with main diet groups centroids (triangles). Ordination plot overlapped with the month contour lines (grey gradient, months indicated on the bottom edge with numbers). Colour of dots indicates rivers.

NMDS ordination into two dimensions resulted in a stress value of 0.18. NMDS plot revealed no clear groups (Fig. 4). No significant differences (p = 0.149) in the overall diet composition between rivers were found, but similarities in the composition of the fish diet were clearly associated with seasonality (Table 2). Diet groups centroids suggested the gradual changes in the fish diet from Hemiptera and Diptera during the summer months, through the terrestrial fauna, then Trichoptera and Ephemeroptera, and Plecoptera in the late autumn. The effect of month was supported by perMANOVA analysis (p = 0.001) together with the effect of fish length (p = 0.009).

**Table 2:** Summary of perMANOVA test based on Bray-Curtis distances of grayling diet composition.

Term	Df	SS	MS	F	R2	P
Length	1	0.636	0.636	2.54	0.02	0.009
River	1	0.378	0.378	1.51	0.01	0.149
Month	6	5.244	0.874	3.49	0.13	0.001
Residuals	134	33.588	0.251		0.84	
Total	142	39.846			1.00	

Df - degrees of freedom, SS - sum of squares, MS - mean sum of squares, F -pseudo-F value, R2 – R-squared value, P – significance level based on 999 permutations.

### Occurrence of the main groups of prey in the diet

**Figure 5:**
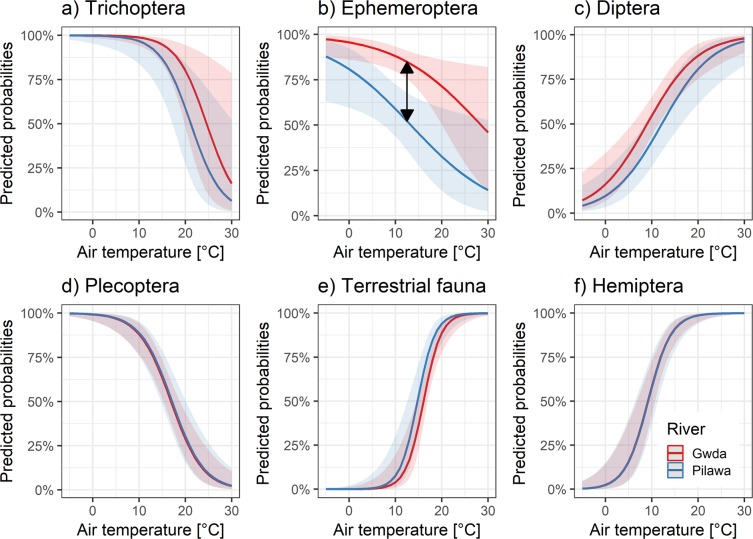
Predictions of the final binomial models fitted with the best signal of air temperature identified with the sliding window analysis. The significant difference in the phenology between rivers is visualized with the arrows. See Supplementary Materials for detailed results of the sliding window analysis and identified critical periods.

The sliding window analysis conducted on the presence-absence data revealed, that the optimal time window of air temperature as a predictor of prey occurrence in the stomach content may vary between different groups of organisms (see Supplementary Materials for detailed results). The effect of air temperature in the identified critical period was negative for Trichoptera, Ephemeroptera and Plecoptera, while positive for Diptera, terrestrial fauna and Hemiptera (Fig. 5a–f), which supports previous results of GLM and NMDS analysis (month effects).

The significant effect of fish length in the air temperature-related GLM binomial models was only found for Hemiptera (Table 3). Analysis of model coefficients revealed a positive relationship between this predictor and response variable (higher probability of Hemiptera occurrence for the larger fish). Significant differences between rivers were found only for Ephemeroptera, suggesting altered phenology for these organisms in the Gwda (Table 3, Fig. 5b). The predicted probability of Ephemeroptera occurrence in the diet is significantly higher for fish from the Gwda than from the Pilawa. Despite the significant effect of air temperature on the probability of Ephemeroptera occurrence in the main model (Table 3), p value obtained for this variable in the randomized permutations was 0.41, showing that the identified temperature signal may not be confident. Randomization tests indicated that the likelihood of obtaining such strong models support by chance was p < 0.05 for all other groups (in most cases p < 0.001, see Supplementary Materials for details).

**Table 3:** Summary of GLM binomial models fitted to the occurrence data with sliding window approach (see Supplementary Materials for details of the sliding window analysis).

Group	Term	LR Chisq	Df	P
Tichoptera	length	0.27	1	0.605
Tichoptera	river	1.38	1	0.239
Tichoptera	air temperature	14.76	1	<0.001
Ephemeroptera	length	0.5	1	0.478
Ephemeroptera	river	14.01	1	<0.001
Ephemeroptera	air temperature	5.93	1	0.015
Diptera	length	0.15	1	0.7
Diptera	river	2.19	1	0.139
Diptera	air temperature	22.28	1	<0.001
Plecoptera	length	0.03	1	0.855
Plecoptera	river	0.03	1	0.859
Plecoptera	air temperature	34.88	1	<0.001
Terrestrial fauna	length	1.57	1	0.211
Terrestrial fauna	river	2.46	1	0.117
Terrestrial fauna	air temperature	27.53	1	<0.001
Hemiptera	length	7.71	1	0.005
Hemiptera	river	0	1	0.957
Hemiptera	air temperature	41.05	1	<0.001

LR Chisq - likelihood-ratio chi square, Df - degrees of freedom, P – significance level.

## Discussion

The grayling occupying mainly upper parts of the rivers^25^ is one of the few predatory fish adapted to collect small drifting macroinvertebrates^17,19^. The results obtained in the study showed that the grayling feed mainly on the aquatic insects. The diet of fish in the Gwda and Pilawa was dominated by Trichoptera, Ephemeroptera and Hemiptera, similar to studies conducted in other water bodies^26–28^. Existing literature indicates the highly opportunistic feeding strategy of grayling, suggesting that species diet reflects the availability of individual food groups in the environment, as in the case of other salmonids^29^. However, analysis of food selectivity conducted in the present study revealed that Ephemeroptera were eaten with high preference, while most of the prey groups were avoided by fish. Moreover, previous studies show that adult grayling feed mainly on benthic invertebrates^19,20,30,31^, while results presented herein suggest that in the systems studied, fish have been able to change their behaviour and feed on the mayfly subimagines floundering on the water surface, together with terrestrial animals which fall into the water. Especially in the Gwda during the late autumn, when the Baetids subimago appeared, a peak in numbers of animals gathered from the surface was observed.

The significant positive effect of fish length in the negative binomial GLM was identified for most of the prey groups. Larger individuals ate macroinvertebrates in larger quantities in order to meet their energetic demands and maintain growth^32^. There was no positive correlation between fish length and abundance of Diptera eaten (dominated by Simuliidae). The proportion of time spent intercepting these tiny prey items and returning to the focal point is unfavourable^32^, therefore the foraging strategy of eating them can be energetically inefficient. These observations confirm previous results, showing an age-related decrease in the percentage of Simuliidae in the diet composition of grayling^27^. No length effect was also found for the Plecoptera group consisting mainly of *L. fusca* and *T. nebulosa*, which, according to the selectivity analysis, were clearly avoided by fish in both rivers.

Strong seasonal patterns of whole diet composition were visible both in the negative binomial GLM models and NMDS analysis. Clear temporal changes were observed in the grayling diet also in the previous studies^27^. Significant effects of the air temperature in the models of prey items occurrence also suggest that the main factor determining the availability of the major feeding groups of the fish is the ambient temperature.

It is assumed that the pattern of grayling feeding is a reflection of the state of the food resources and can considerably differ even in neighbouring areas^26^. However, NMDS analysis followed by perMANOVA showed no river effect, suggesting the consistent composition of a whole community of macroinvertebrates in the grayling diet in both locations. At the same time, GLM tests showed significant differences in the abundances and probability of occurrence of Ephemeroptera in the two studied rivers. Higher abundances of Ephemeroptera in the Gwda were associated mainly with the emergence of *B. liebenauae*, which peaks during the colder period of the year in October and November. It has been shown by the previous work^8^ and confirmed in the present analysis that water reservoirs built along the Gwda altered water temperature. Furthermore, it has been concluded in the previous study that higher temperatures of water might postpone diapause termination of mayflies which would lead to a later onset of the development of the last generation, thus the emergence of a second generation of the mayflies is delayed and reduced by the later river cooling^8^. These findings were supported by the presented binomial GLM model showing a higher probability of Ephemeroptera occurrence in the grayling diet in the river Gwda and significant phenological shift of these organisms (no differences in the phenology between rivers were found for other groups).

The air temperature was identified as a significant predictor of occurrence for the majority of groups of organisms in the grayling diet. Sliding window analysis revealed that the strongest environmental signal for the emergence of multivoltine insects (which have several generations per year), like Trichoptera, Ephemeroptera or Diptera come from the period just before appearing in the fish diet. In the temperate regions, these short-lived organisms respond abruptly to the environmental signals (mainly day-length and ambient temperature) in order to take advantage of favourable conditions and to complete their life cycles^33^.

The changed temperature in the Gwda distorted the quality of information given by environmental cues that mayflies use to make developmental decisions^8^. This led to the developmental trap, a special case of evolutionary trap^9,10^. Interestingly, despite the significant effect of air temperature in the main developed GLM binomial model, randomization tests did not support this environmental signal as a predictor of Ephemeroptera occurrence in the fish diet (while probabilities obtained for other taxons were highly significant). These results support previous findings, suggesting a blurred environmental cue experienced by the population of mayflies in the Gwda^8^. These discrepancies between signals reflected by water temperature and environmental conditions associated with air temperatures are likely to be detrimental to mayflies because the postponed late-season phenology resulted in the suicidal last generation^8^. In the system of the Gwda river, the nymphs develop in the more favourable conditions in the water but often reach maturity when the conditions in the air became, in contrast, suboptimal or adverse for flying. Their response to the water temperature changes trapped them, reducing their reproduction. In the Gwda, typical evolutionary traps^11^ appeared.

The “lost generation”^9^ of mayfly which failed to complete development, may be consumed and can favour the fish from altered watercourses. During colder months (October and November) they can constitute a valuable source of energy for grayling, stimulating alternative feeding on the surface of the water. Thus, it can improve their condition, especially when it happens just before the winter, a few months before spawning. Increased availability of this food resource may cause also further implications for the fish population dynamics, which were not studied in the present work. For this reason, additional long-term studies are needed to determine the net benefit of increased consumption of the mayflies by drift-feeding fish, both on the individual (e.g. condition factor) and population level (e.g. biomass of stock, accounting for fish stocking and fishing harvest). Moreover, because the study period was restricted to the second half of the year (June-December), further investigations covering the whole year would be of great advantage for a more complete understanding of trophic interactions between drift-feeding fish and macroinvertebrates in different stages of their life cycle. Because only two (one natural and one anthropogenically impacted) rivers were surveyed in the present study, the larger number of sites and samples, and a better control of other environmental parameters potentially influencing predator-prey interactions (e.g. current speed, chemistry of water) are needed in order to achieve more reliable results and stronger conclusions about mechanisms of the investigated processes. The application of more non-selective types of sampling equipment is also advised, because of potential constraints of the fly fishing methods used, which can to some extent bias the results of the diet composition analysis. However, in this study attention was paid during sampling design to maximize randomness and representativeness of fish samples. Different fishing methods, covering surface, central part of the water column and bottom layer were applied in order to reduce the selectivity of individuals feeding only in a specific zone or on a particular type of food. The results presented are comparable with previous studies, where the same sampling methods were applied^34,35^, but caution should be made during interpretation.

The detailed description of consequences of altered river thermal conditions, which directly disturb the population dynamics of macroinvertebrates, but also through these changes indirectly affect fish located on the higher trophic levels is of high importance for our understanding of the potential climatic effects on feeding ecology of freshwater fish^22^. The results presented, indicate that reservoir-induced thermal alterations in the rivers, similarly to climate change, can lead to a chain of consequences and disturb previous predator-prey interactions in the water ecosystems. Taking into consideration the projected climate scenarios^2,24^, further monitoring and forecasting of these effects are considered an important step for future mitigating actions and adaptive management of water resources^23^.

## Materials and Methods

### Study area

Two closely located rivers in the central Europe (North West Poland) were selected to carry out the field observations. They have similar characteristics and catchment area (Table 4). The main sampling site was located on the river Gwda, 7 km below the dam, where the water temperature was elevated by numerous reservoirs by 0.5–2.0 °C during the summer^8^ (Fig. 1). A changed thermal regime of the ecosystem prolongs summer diapause of the dominating mayfly species (*B. liebenauae*) in Gwda, shifts its lifecycle and by altering phenology results in a largely lost autumn generation of these insects^8^. The second study site was located on a river, which is characterized by natural, undisturbed flow (thus, treated as the control). River Pilawa is the biggest tributary of the Gwda. The study site was in the middle part of the watercourse. The smaller width of the river created a channel that was divided into parts shaded by alder or flowing through meadows which were without a canopy and were overgrown by macrophytes. The assemblages of fishes and invertebrates were similar in both two rivers. They are occupied by the cold-water fish, brown trout (*Salmo trutta*) and grayling, and a macroinvertebrate fauna dominated by mayflies and caddisflies.

**Table 4:** Rivers characteristic (a) and sample size (b).

River	Gwda	Pilawa
**(a) characteristic**		
Catchment [km^2^]	4,947	1,352
Length [km]	145	82
Width* [m]	30	15
Depth* [m]	0.8	0.6
Slope [‰]	0.8	0.4
Current* [m/s]	0.5	0.6
Discharge [m^3^/s]	27	8
**Main type of bottom substratum**	**gravel, boulders**	**gravel and sand**
**(b) sample size**		
N-fish: diet composition	96	47
N-fish: food selectivity	26	14
N-drift samples	13	10

Maximal values were given where indicated (*).

### Fish sampling and stomach content analysis

Fish (143 individuals) were sampled with fly fishing from June to December in the years 2005–2016 (Table 4). Since fly fishing can be selective for the larger individuals, the total length of fish was measured to the nearest cm and incorporated in the analysis in order to control for possible length-related bias in the diet composition. Randomness and representativeness of fish diet samples were maximized by the application of different fishing methods, covering surface, central part of the water column and bottom layer. Stomachs of fish were removed and preserved in alcohol (70% ethanol) and the contents analysed in the laboratory. All the food items found were identified to the lowest possible taxonomic level, considering also the developmental stage of the individuals. The abundance of detritus and vegetal rests was not quantified because it was impossible to count individual items^36^. Organisms were further pooled into five operational taxonomic units^37^ covering the main insect orders. Terrestrial fauna and other prey items, which occurred occasionally, were pooled into two additional categories.

### Sampling of drifting macroinvertebrates

Sampling of drifting macroinvertebrates was conducted in both rivers in the years 2008–2010 during fishing surveys (Table 4). Drift nets of 1 m width and mesh size 1 mm were placed for 5 to 8 hours during the day (depending on the length of the daylight period) in the shallower sites to sample organisms drifting in the whole water column (covering bottom, middle water and surface). All organisms were preserved in 70% ethanol and further identified in the laboratory using the same procedure as during fish stomach content analysis.

### Temperature data

Water temperature data complementary to that presented in the previous work^8^ were recorded using i-Button loggers in the main site on Gwda (below dams) and in the additional location above the artificial dams (Fig. 1). Six temperature measurements during the day (every 4 hours) were recorded in 2010 from 1^st^ of August to 31^st^ of November. Air temperature data were obtained from E-OBS v 17.0 gridded dataset in order to evaluate the climate signal on the occurrence of different prey items in the diet of grayling. This dataset provided a daily high-resolution (0.25° cell) mean surface temperature interpolations for the period from 1950 to the present^38^. Daily mean values of air temperature were aggregated over the area 16.25–17.00°E; 53.25–53.5°N covering both the two sampling sites (Fig. 1).

### Statistical analysis

Differences in the water temperature above and below dams were tested with generalized linear model (GLM)^39^ including water temperature as response variable and location and month as categorical fixed effects, allowing for their interactions. Identity-link function and Gaussian error distribution were assumed in the test.

To analyse the diet composition of fish, percentage numerical abundance of a given prey group and its frequency of occurrence were calculated with the following formulae:

$${C}_{i}=\frac{{n}_{i}}{{\sum }_{i=1}^{m}{n}_{i}},$$

$${F}_{pi}=\frac{{N}_{1i}}{{N}_{p}},$$

where *C*_*i*_ is the percentage numerical abundance, *n*_*i*_ is the number of *i*th food item and *m* the number of food items, *F*_*pi*_ is the frequency of occurrence, *N*_1*i*_ is the number of stomachs in which the food item *i* was found and *N*_*p*_ is the number of non-empty stomachs^40^.

The selectivity of grayling feeding on each group was quantified with Ivlev’s selectivity index (E)^41^. Selectivity was assessed for 39 fish individuals sampled in the years 2008–2010 for which both diet composition data and associated macroinvertebrates drift data (abundances of different prey groups in the environment) were available. The index was calculated with the following formula:

$${E}_{i}=\,\frac{{r}_{i}-{p}_{i}}{{r}_{i}+{p}_{i}},$$

where *r*_*i*_ is the relative abundance of food category *i* in the stomach (as a proportion or percentage of all stomach contents) and *p*_*i*_ is the relative abundance of this prey in the environment^42^. Values of this index range from −1 to +1, with negative values indicating rejection or inaccessibility of the prey, zero indicating random feeding, and positive values indicating active selection^36^.

Further, a series of GLM were fitted to the data to evaluate different sources of variation in abundances of main prey groups (excluding group “other”). Group “other” was excluded from the analysis because it constituted a mixture of different organisms, which were not linked systematically or functionally, limiting the reliable ecological inference. After preliminary tests, negative binomial distribution and log-link function were used in the modelling^43^. The abundance of a given prey group was treated as a response variable. Month and river were included in the models as fixed factors and fish length as a covariate. A single observation from June was excluded before the analysis.

Non-metric multidimensional scaling (NMDS) analyses were applied in order to visualize variation in the whole composition of grayling diet^44^. Bray–Curtis dissimilarity index of fish diet composition by groups and Wisconsin standardization with square root transformation were used to ensure a more uniform basis for comparison of diet matrix. Each observation was plotted in the two-dimensional space showing taxonomic dissimilarities among the diet of fish individuals. Centroids of main diet groups were overlaid on ordination together with the smooth surface of month variable to assess temporal changes in the composition of the fish diet. Following multidimensional scaling, permutational multivariate analysis of variance (perMANOVA) was carried out with 999 permutations using distance matrix^45^. Similarly, to the previous GLM tests, month and river were included in the models as fixed factors and fish length as a covariate.

In the natural river systems, air and water temperatures are known to be closely related and constitute predictor of macroinvertebrates phenology^46–48^. Assuming the opportunistic feeding strategy of grayling, we expected, that appearance of prey group in the environment translates to its occurrence in the diet, therefore fish diet composition may be predicted with air temperature. Phenological differences between sites may be associated with alterations in the ecosystem (e.g. changes in water temperature caused by reservoirs), thus help to evaluate their possible consequences for fish trophic interactions. In order to test this hypothesis and to assess the influence of local air temperatures on the occurrence of each main diet group, separate analyses were conducted using the GLM framework. The binomial model with logit-link function was used for diet data translated into presence/absence. Occurrence binary data were treated as response variables. The length of fish was included in the baseline models as a covariate in order to consider possible shifts in the prey preferences during the fish ontogeny. The river term was included as a fixed factor to evaluate different phenological responses between sites. The month variable was excluded from this part of the analysis to avoid covariation with temperature^8^. Appearance of each group of organisms constituting diet of grayling may be affected by the ambient air temperature within different periods (varying durations and time lags)^49^, therefore series of sliding window analyses^50,51^ were conducted on the biological and climatic data from the years 2005–2016 in order to identify the optimal time window of the air temperature predictor (see Supplementary Materials for details).

All models were checked for overdispersion and residuals were checked for homoscedasticity and normality. The significance of variables was assessed with p-values generated using log-likelihood ratio comparisons. Significance was accepted at p < 0.05. All data were analysed using *R* scientific computing language^52^. Negative binomial models were fitted with the *glm.nb* function of *MASS* package^53^. NMDS was conducted with *metaMDS* function, smooth surface was fitted with *ordisurf* function and perMANOVA run with *adonis* function of *vegan* package^54^. Sliding window analysis was carried out with *climwin*^55^. Package *car*^56^ was applied for significance tests and package *tidyverse*^57^ for data visualization.

### Ethical statement

All the fish used in the study were collected with the licenses (No. 1634, 493) and permits issued by the Polish Angling Association in accordance with relevant guidelines and regulations (The Inland Fisheries Act, The Official Journal of Laws of the Republic of Poland of 1985, No. 21, Item 91, and the subsequent updating).

## Supplementary information

Supplementary materials
