# Supplementary material for: Cascading effects of temperature alterations on trophic ecology of European grayling (Thymallus thymallus)
Source: Sci Rep. 2019 Dec 4;9:18358. doi: 10.1038/s41598-019-55000-5 (PMC6892815; doi:10.1038/s41598-019-55000-5)
Supplement: Supplementary file 1 — Supplementary materials [file 41598_2019_55000_MOESM1_ESM.pdf]

Supplementary materials:

Cascading effects of temperature alterations on trophic ecology of European grayling (*Thymallus thymallus*)

Szymon Smoliński[1,2*], Adam Glazaczow[3]

[1]Department of Fisheries Resources, National Marine Fisheries Research Institute, Kołłątaja 1, 81-332 Gdynia, Poland

[2]Demersal Fish Research Group, Institute of Marine Research, P.O. Box 1870 Nordnes, 5817 Bergen, Norway

[3]Department of Systematic Zoology, Adam Mickiewicz University, Umultowska 89, 61-614 Poznan, Poland

*corresponding author, tel.: +48 587-356-193, e-mail: ssmolinski@mir.gdynia.pl

Comparison of the water temperature above and below dams

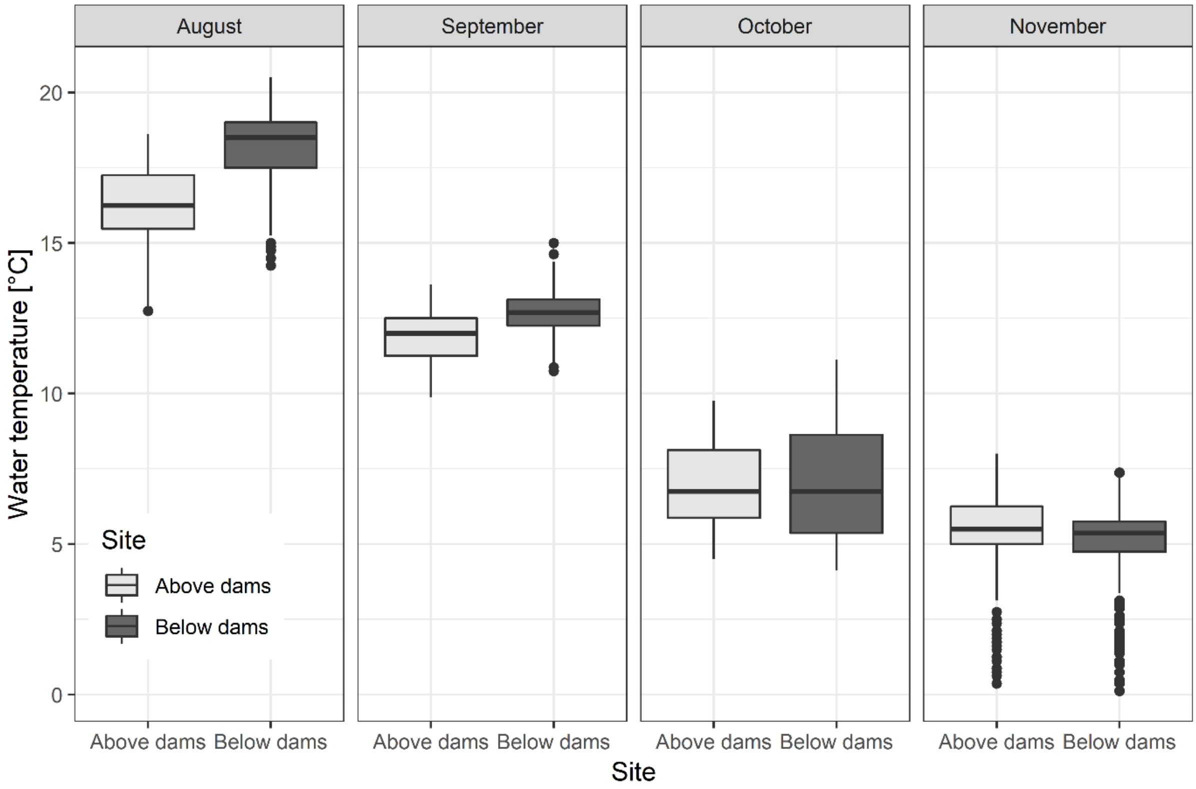

Fig. S1. Mean daily temperatures observed above and below dams built along course of Gwda. Lines, boxes, and whiskers are medians, interquartile range (IQR), and 1.5× IQR, respectively.

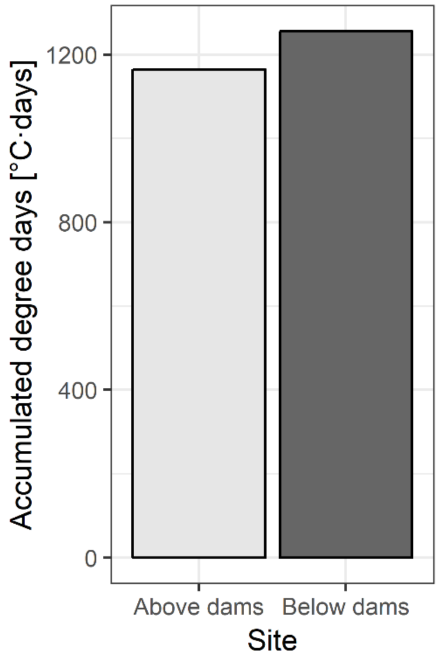

Fig. S2. Accumulated degree days above and below dams built along course of Gwda. Mean daily temperatures are accumulated for the 1st August – 31st October.

Tab. S1. Summary of GLM model fitted to the water temperature data recorded in the period 1st of August to 31st of November (N=1426). CI- confidence intervals, P – significance level.

Terms	Estimates	CI	P
(Intercept)	16.16	15.94 – 16.38	<0.001
September	-4.26	-4.56 – -3.96	<0.001
October	-9.16	-9.46 – -8.85	<0.001
November	-11.02	-11.32 – -10.72	<0.001
Below dams	1.92	1.61 – 2.22	<0.001
September: Below dams	-1.14	-1.57 – -0.71	<0.001
October: Below dams	-1.84	-2.26 – -1.41	<0.001
November: Below dams	-2.09	-2.52 – -1.66	<0.001

Sliding window analysis

This analysis allows to identify the optimal time window of air temperature in prediction of different prey groups occurrence in the grayling diet. Baseline models (only fish length and river effect included as explanatory variables) were compared with models that take into account also mean value of temperature from different time windows (various start dates and the durations). The strength of short-term environmental signals was evaluated using Akaike Information Criterion corrected for the small sample sizes (AIC_c) within the range of 100 days before the sampling occasion. Relative time windows (counted "back" from the date of fish sampling) were used to aggregate temperature information and the linear relationship between response and environmental variable was assumed.

The risk of false positives where spurious relationships are interpreted as true environmental signals occurs because of the exploratory nature of sliding window analysis and high number of windows tested during analysis. In order to evaluate the probability of false positives 500 randomization tests were conducted by reshuffling the date variable in the original response data frame, removing any relationship between climate and the biological response. Such approach helps to preserve any relationship between the response variable and other covariates and maintain autocorrelation within the climate data. The distribution obtained of the AIC_c values of the best model selected in each random iteration (with no environmental signal) was compared with AIC_c of the final model to calculate the probability of observed results being the product of chance.

The best predictors of Trichoptera and Ephemeroptera occurrence in the grayling diet have a relatively short-term lag of 5 or 7 days before fish sampling, respectively (Tab. S2). For these two groups the environmental signal was clearly concentrated within the last days before sampling (Fig. S3a, S3b), while for Diptera, Plecoptera and Hemiptera, the regions of significant air temperature effects are extended over several days (Fig. S3c, S3d, S3f). The best-

supported time windows during which mean air temperature affect occurrence of Diptera, Plecoptera and Hemiptera in the grayling diet were 17-12, 43-42 and 43-20 days before sampling occasion, respectively (Tab. S2). Air temperature from the period 99-58 days before observation was considered an optimal environmental predictor of occurrence of terrestrial fauna in the stomach content (Tab. S2, Fig. S3e).

Tab. S2. Results of sliding window analysis for air temperature as predictor of occurrence of main diet groups. ΔAIC_c – difference in AIC_c of baseline model and model with included air temperature effect, P – probability from 5000 randomization tests (see. Fig. S4).

Group	ΔAIC_c	Window Open	Window Close	P
Trichoptera	-12.64	5	5	0.010
Ephemeroptera	-3.81	7	7	0.410
Diptera	-20.16	17	12	<0.001
Plecoptera	-32.77	43	42	<0.001
Terrestrial fauna	-25.41	99	58	<0.001
Hemiptera	-38.94	43	20	<0.001

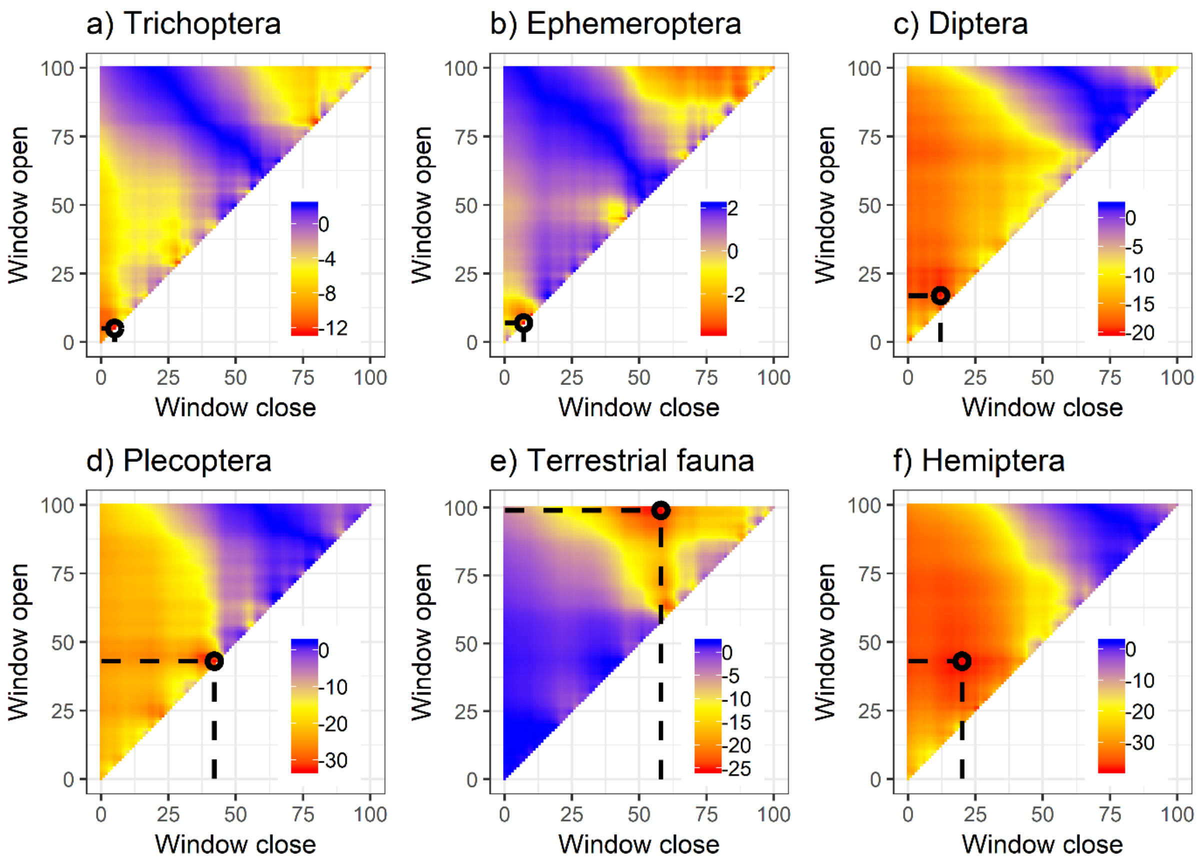

Fig. S3. The results of optimal time window identification for the air temperature as predictor of grayling food groups occurrence. The days of window open and close (counted back from the day of fish sampling) are shown on the axes. ΔAIC_c (indicated by the gradient) are the differences in AIC_c between the base model and model with included climatic effect (air temperature).

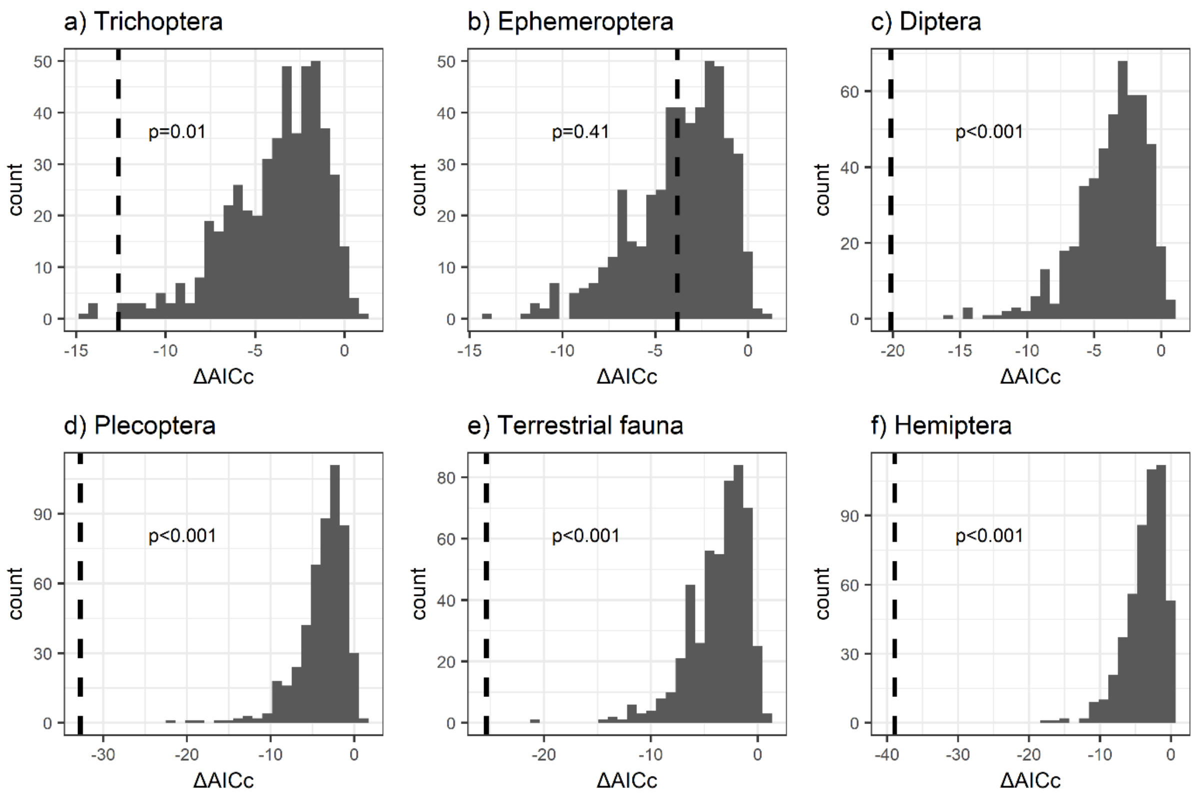

Fig. S4. Results of 500 randomization tests for optimal time window identification on occurrence data. Histograms present distribution of the best AIC_c obtained during sliding window analysis on datasets with the reshuffled date variable in the original response data frame (removing any relationship between climate and the biological response). Vertical dashed lines indicate the final AIC_c results obtained during the sliding-window analysis on the original datasets.
